# Clinical Isolation of *Anaplasma phagocytophilum* in South Korea

**DOI:** 10.4269/ajtmh.16-0529

**Published:** 2017-10-30

**Authors:** Seung Hun Lee, Se Yoon Park, Mi Jin Jang, Ki Ju Choi, Hae Kyung Lee, Young-Uk Cho, Yeong Seon Lee, Sung-Han Kim, Seon Do Hwang

**Affiliations:** 1 Division of Zoonoses, Center for Immunology and Pathology, Korea National Institute of Health, Korea Centers for Disease Control and Prevention, Chungcheongbuk-do, Republic of Korea;; 2 Department of Infectious Diseases, Asan Medical Center, University of Ulsan College of Medicine, Seoul, Republic of Korea;; 3 Division of Biosafety Evaluation and Control, Korea National Institute of Health, Korea Centers for Disease Control and Prevention, Chungcheongbuk-do, Republic of Korea;; 4 Department of Laboratory Medicine, Asan Medical Center, University of Ulsan College of Medicine, Seoul, Republic of Korea;; 5 Division of Infectious Diseases, Department of Internal Medicine, Soonchunhyang University Seoul Hospital, Soonchunhyang University College of Medicine, Seoul, Republic of Korea

## Abstract

We report the first isolation of *Anaplasma phagocytophilum* in South Korea. A 61-year-old woman presented with a 6-day history of fever, headache, and myalgia. Initial investigation showed neutropenia and thrombocytopenia. We diagnosed human granulocytic anaplasmosis by microscopic examination and serologic testing. The patient recovered fully without antibiotic therapy. The isolate was obtained from the patient’s blood by cell culture and mouse inoculation. Its identity was confirmed by an immunofluorescence assay, sequencing of the 16S rRNA gene, *msp2* (*p44*), and *ankA* genes, and staining and electron microscopy of morulae of *A. phagocytophilum* in cultured human promyelocytic leukemia HL-60 cells.

## INTRODUCTION

The obligate intracellular bacterium *Anaplasma phagocytophilum* causes human granulocytic anaplasmosis (HGA). The bacterium is a zoonotic tick-borne pathogen mainly transmitted by *Ixodes* spp. ticks that infect domestic mammals and humans.^1^ Clinical features of the human infection range from mild illness such as fever, headache, myalgia, malaise, thrombocytopenia, and leukopenia to severe disease with gastrointestinal and respiratory distress, myocarditis, neurological complications, septic shock-like disease, and even death.^2,3^ However, the majority of human infections caused by *A. phagocytophilum* do not result in severe disease.^4^

In South Korea, *Ixodes* spp. ticks are uncommon.^5^ However, *A. phagocytophilum* has been demonstrated in *Haemaphysalis longicornis* ticks, which are the most abundant species in South Korea,^6^ and this has led to growing concern about the possible emergence of HGA in South Korea. Actually, recent seroprevalence studies have shown that 1.8% of serum samples from febrile patients were positive for *A. phagocytophilum* in an immunofluorescence assay (IFA) test in 2002, and in 2003 the percentage was 8.9% from patients with symptoms of high fever suspected mainly scrub typhus.^7,8^ Despite this, the first HGA case with isolation in South Korea was only reported in 2014^9^ and there were no other reports of isolation of *A. phagocytophilum* from the patients. Here, we report the second and better defined isolation of *A. phagocytophilum* from a patient with HGA.

## STUDY

**Figure 1.**
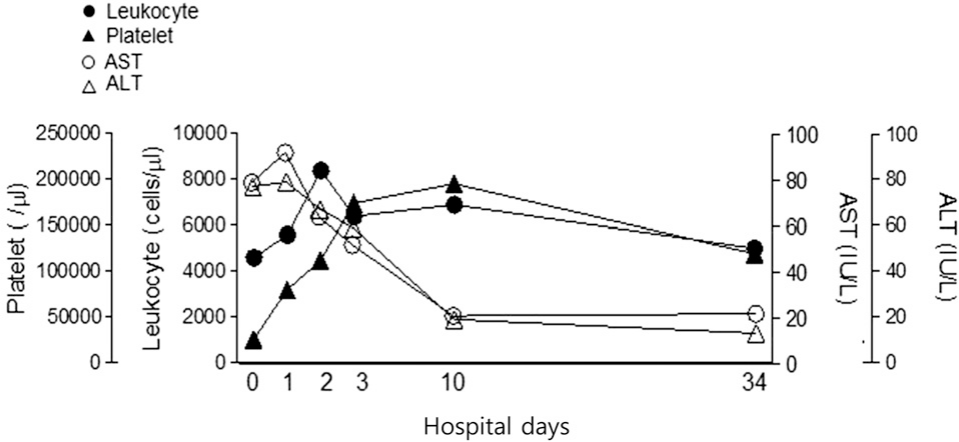
Time-course of white blood cells (WBCs), platelets, aspartate aminotransferase (AST), and alanine aminotransferase (ALT) in the human granulocytic anaplasmosis patient.

A 61-year-old woman presented to a hospital in Jeonju City with 6-day fever, headache, and myalgia on May 19, 2015. She lived in Jeonju City, province of Jeollabuk-do, Korea. She had visited her ancestor’s grave and there, she had gathered bracken on a hill about 3 weeks earlier. She was transferred to Asan Medical Center because of bicytopenia (white blood cells 2.9 × 10^9^/L and platelets 9.0 × 10^9^/L). Body temperature was 36.6°C, blood pressure 107/72 mmHg, and pulse rate 84 beats per minute. Headache and chill gradually improved from the time of admission. There was no eschar or skin rash. Laboratory tests showed pancytopenia and poor liver function (Figure 1). The clinical condition of the patient improved rapidly without antibiotics; she recovered fully by hospital day 4 and was discharged. She was followed as an outpatient at month later. The bicytopenia fully resolved (Figure 1).

**Figure 2.**
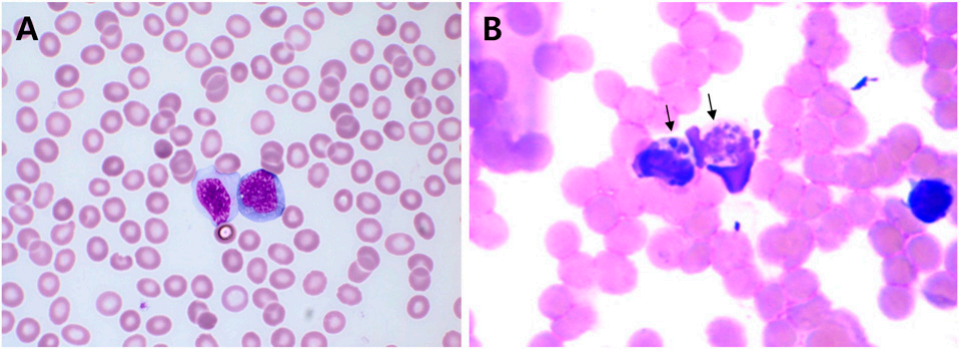
(**A**) A peripheral blood smear showing bicytopenia with occasional circulating reactive lymphocytes (Wright–Giemsa stain, ×1000), thin smear; (**B**) *Anaplasma phagocytophilum* morulae (*arrows*) in peripheral white blood cells (**A**; Diff-Quik staining) of patient’s blood. Original magnification, ×400.

The initial differential diagnosis was severe fever with thrombocytopenia syndrome, a rickettsial disease such as HGA, scrub typhus, or possible hematologic malignancy such as leukemia. Tests for hantavirus, *Leptospira*, and *Orientia tsutsugamushi* antibodies were negative. Peripheral blood smear demonstrated mild anemia and marked thrombocytopenia with increase of reactive lymphocytes. No *A. phagocytophilum* morulae were detected (Figure 2A). A bone marrow biopsy was performed to differentiate hematologic malignancy, but there was no evidence of hematologic disease, except for occasional occurrence of hemophagocytic histiocytes. However, we found basophilic inclusions in vacuoles suggestive of histiocytes containing *A. phagocytophilum* morulae (Supplemental Figure 1).

**Figure 3.**
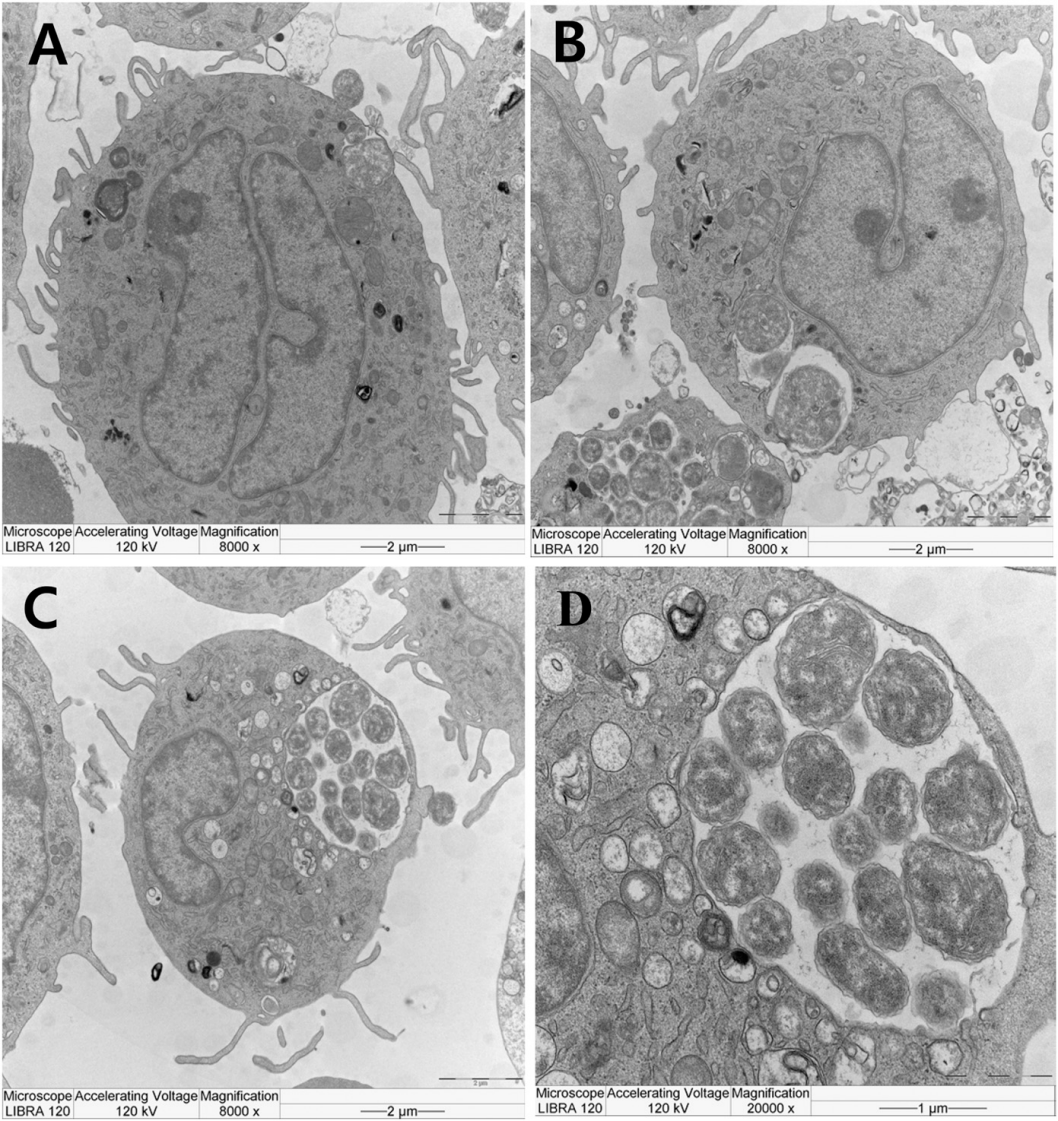
Adhesion, invasion and replication of the isolated *A. phagocytophilum* within HL-60 cells. *Anaplasma phagocytophilum* in mouse spleen extract were incubated with HL-60 cells (**A**: adhesion, **B**: invasion, **C** and **D**: replication).

To observe bacteria within monocytes and neutrophils, a thick peripheral blood smear was stained with Diff-Quik solution. *Anaplasma phagocytophilum* morulae were found within the monocytes (Figure 2B). We confirmed that *Ehrlichia chaffeensis* was not detected by specific 16S rRNA gene nested polymerase chain reaction (PCR) in patient blood and IFA using a commercial kit revealed it was not *E. chaffeensis*. To amplify the *A. phagocytophilum* from blood we inoculated the buffy coat of the 100 µl of buffy coat from patient’s blood into two C3H/HeJ mice via intraperitoneal route.^10^ Protocols using live animals were reviewed and approved by an Institutional Animal Care and Use Committee (KCDC-049-15-1A). On day 7 postinoculation (dpi7), we obtained a positive result for IFA against *A. phagocytophilum*, and a positive result for PCR of the 16S rRNA gene in infected mouse blood on dpi 10.^11^ On dpi 14, the infected mice were killed and we observed splenomegaly (Supplemental Figure 2). To culture the *A. phagocytophilum*, cells of the human promyelocytic leukemia cell line HL-60 (KCLB-10240) were inoculated with a homogenate of infected mouse spleen in RPMI1640 medium (Thermo Fisher, Waltham, MA) supplemented with 2% fetal bovine serum (Thermo Fisher) and 2 mM L-glutamine (Thermo Fisher) and cultured in an incubator at 37°C with 5% CO_2_ with regular change of medium (cell density of 1–5 × 10^5^ cells/mL). The cultured cells were examined microscopically by Diff-Quik staining of cytocentrifuged cells at 2–3 day intervals. On dpi13 we observed morulae within the cultured HL-60 cells (Supplemental Figure 3), and transmission electron microscopy revealed adhesion and invasion of individual *A. phagocytophilum* and their replication within the HL-60 cells (Figure 3). DNA was extracted from initial patient’s blood with a G-spin Total DNA Extraction Mini kit (Intronbio, Korea) to detect *A. phagocytophilum* 16S rRNA, *ankA*, and *msp2* DNA.^11–13^ Nested PCR was conducted using *A. phagocytophilum*-specific primers to amplify fragments of the 16S rRNA, *ankA*, and *msp2* genes.^11–13^ This gave positive results for the infected cells as well as the patient’s blood. Direct sequencing of the PCR products confirmed the isolate as *A. phagocytophilum* (NCBI accession Nos. 16S rRNA:KT986058, *ankA*:KT986059, *msp2*:KT986060).

**Figure 4.**
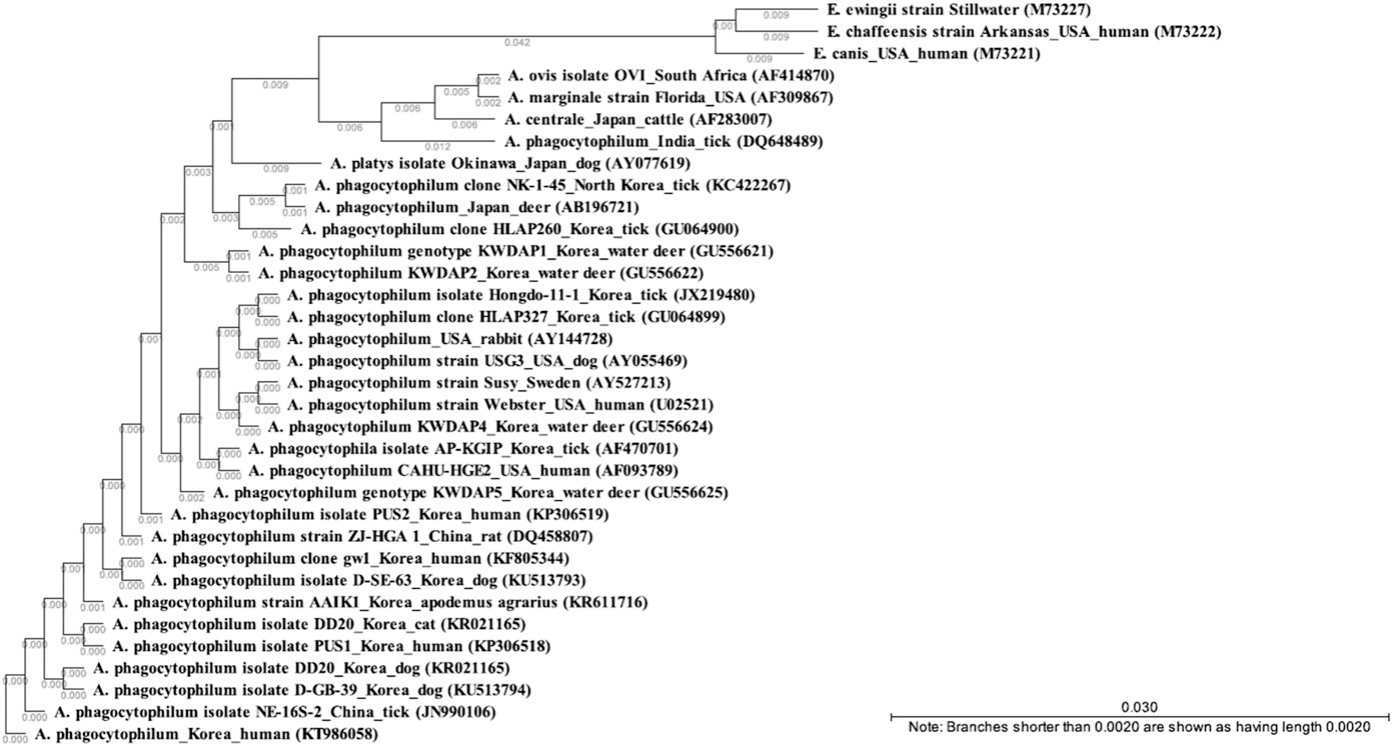
Phylogenetic tree of the 16S rRNA gene sequence of *Anaplasma phagocytophilum* isolated from the patient’s buffy coat. The tree was constructed by the neighbor-joining method. Scale bars indicate sequence distances.

The PCR sequences obtained from the isolate were 99–100% similar to *A. phagocytophilum* for the 16S rRNA, especially with Korea (2014, human, KF805344). The 16S rRNA sequences were dissimilar to the isolates from United States and Sweden (Figure 4). In addition, the sequences of *ankA* and *msp2* of our isolate were distant from those of strains isolated in other countries from patients, ticks, and animals, but similar to other Korean isolates. In terms of serology, seroconversion was observed using a commercial *A phagocytophilum* serologic kit (Fuller Laboratories Fullerton, CA): the patient’s serum was negative on day 4 but by day 37 it had increased to IgG 640 and IgM 16.

## CONCLUSIONS

We have obtained a Korean *A. phagocytophilum* isolate through animal experiment and cell culture using a blood specimen from a patient who had spent time on a mountain. In a phylogenetic analysis, this isolate was similar to other Korean *A. phagocytophilum* isolates with 100% identity in the investigated region of the 16S rRNA gene (Figure 4). There have been a few reports of serological surveys and molecular surveys in ticks, animals, and humans in Korea. Using molecular detection methods, 9.9% ticks were found infected in 2003,^6^ 63.6% in Korean water deer in 2011^14^ and by serological detection 1.8–8.9% was shown in humans in 2002–2003.^7,8^ Although there has been concern about the emergence of HGA in South Korea, to our knowledge this is the second case of HGA in South Korea following the first patient.^9^ In addition, this is an extant clinical isolate has been obtained from an infected Korean patient, and this will provide a basis for characterizing a human isolate and identifying diagnostic materials in South Korea. Furthermore, this human isolate may play a role as a reference strain for studying transmission and pathogenicity using Korean ticks and other animals.

This case shows that detecting *A. phagocytophilum* morulae in thin peripheral blood smears is difficult in actual clinical practice. The detection rate of morulae was known to be 25–75%, and is higher in the first week of a disease than in later weeks.^2^ We performed microscopic examination through whole blood for further experimental analysis. No morulae were found in a thin blood smear from the patient at 7 days from the development of fever and myalgia (Figure 2A) but morulae were found in a thick smear (Figure 2B). Recently, it has been suggested that inspection of more than 200 granulocytes could increase the diagnostic yield.^15^ We therefore suppose that there are not enough granulocytes to detect morulae in the thin smears routinely used to evaluate hematologic disorders. However, thick smears can permit examination of more than 500 granulocytes. So, we assume that thick blood smears would be more sensitive than thin blood smears in patients with suspected HGA. Furthermore, blood smear using buffy coat may increase the sensitivity for the detection of morulae, although we did not perform blood smear this way. In addition, we obtained a negative serologic result on day 4 from the onset of fever, but found an IgG titer of 640 one month later. This emphasizes that paired samples are needed for serologic confirmation. We conclude that although the detection rate in morulae will vary according to granulocyte density and may be affected by interobserver variability, the use of thick and thin blood smear evaluation could be helpful, especially in the acute phase of the illness. Until more data are available, in the early stage of HGA, molecular diagnosis by PCR and blood smear evaluation are the preferred procedures for diagnosing HGA.

## Supplementary Material

Supplemental Figure.
