# Supplementary material for: Clinical Isolation of Anaplasma phagocytophilum in South Korea
Source: Am J Trop Med Hyg. 2017 Oct 30;97(6):1686–90. doi: 10.4269/ajtmh.16-0529 (PMC5805025; doi:10.4269/ajtmh.16-0529)
Supplement: Supplementary file 1 [file tpmd160529.SD1.pdf]

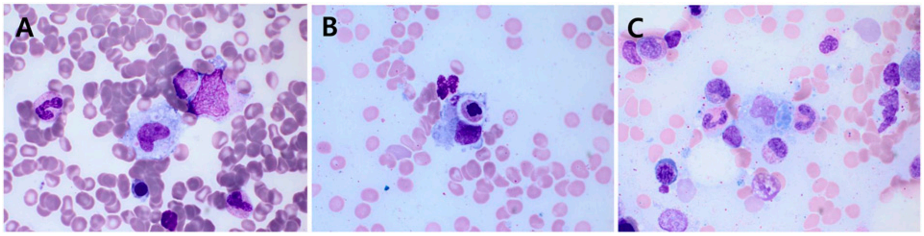


Supplemental Figure 1. An aspirate smear showing hemophagocytic histiocyte-engulfing platelets or neutrophils as well as a histiocyte containing mulberry-like intravacuolar inclusions suggestive of morulae of *Anaplasma phagocytophilum* (Wright–Giemsa stain, ×1000).

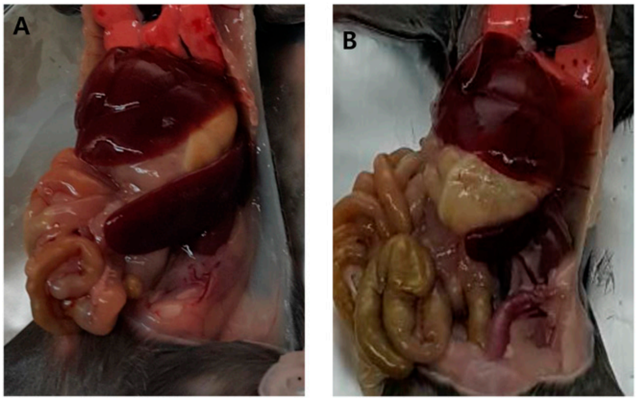


Supplemental Figure 2. Spleen of a C3H/HeJ mouse infected with the patient's buffy coat; day 14; splenomegaly. The spleen is twice as large as normal.

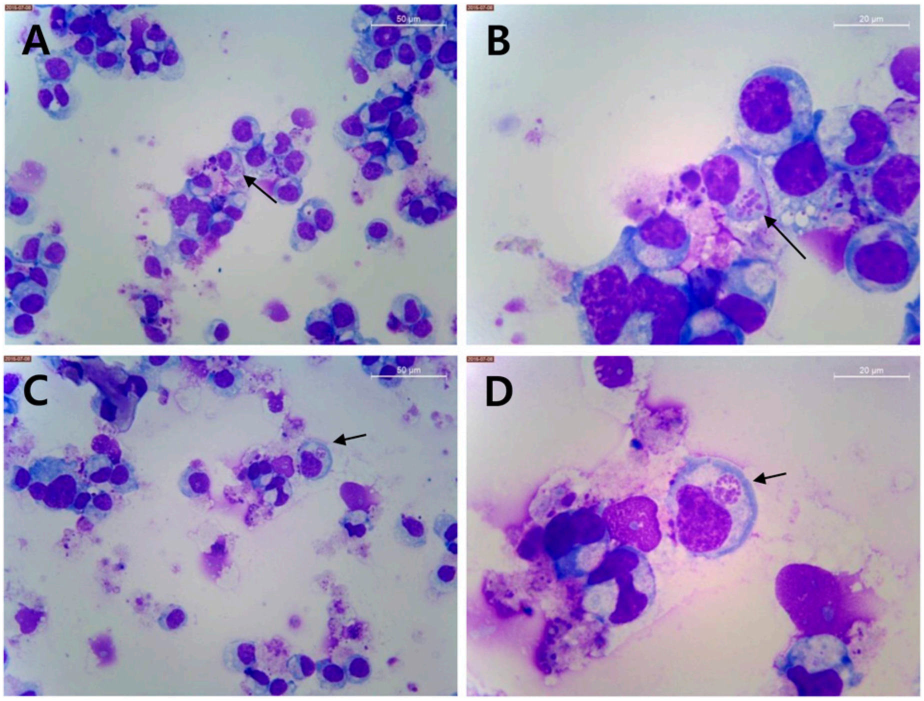


Supplemental Figure 3. Light micrograph of *Anaplasma phagocytophilum* cultured in a human promyelocytic cell line (**A**–**D**; Diff-Quik staining). Original magnification (**A** and **C**; ×400, B and D; ×1,000).
